# Childhood adversity predicts striatal functional connectivity gradient changes after acute stress

**DOI:** 10.1162/imag_a_00269

**Published:** 2024-08-19

**Authors:** Xiang-Shen Liu, Koen V. Haak, Karolina Figa, Janna N. Vrijsen, Marianne Oldehinkel, Peter C.R. Mulders, Rose M. Collard, Philip F.P. van Eijndhoven, Christian F. Beckmann, Guillén Fernández, Indira Tendolkar, Nils Kohn

**Affiliations:** Department of Cognitive Neuroscience, Donders Institute for Brain, Cognition and Behaviour, Radboud University Medical Centre, Nijmegen, The Netherlands; Department of Cognitive Science and Artificial Intelligence, Tilburg University, Tilburg, The Netherlands; Department of Psychiatry, Donders Institute for Brain, Cognition and Behaviour, Radboud University Medical Centre, Nijmegen, The Netherlands; Pro Persona Mental Health Care, Depression Expertise Center, Nijmegen, The Netherlands; Department of Psychiatry, Radboud University Medical Centre, Nijmegen, The Netherlands; LVR-University Hospital Essen, Department of Psychiatry and Psychotherapy, Medical Faculty, University of Duisburg-Essen, Essen, Germany

**Keywords:** childhood adversity, acute stress, striatum, connectopic mapping

## Abstract

As a primary risk factor for psychiatric vulnerability, childhood adversity (CA) leads to several maladaptive behavioral and brain functional changes, including domains of emotion, motivation, and stress regulation. Previous studies on acute stress identified the potential role of a striatum-centered network in revealing the psychopathology outcomes related to CA. To elucidate the interplay between CA, acute stress, and striatal functions in psychiatric disorders, more evidence from large-scale brain connectivity studies in diverse psychiatric populations is necessary. In a sample combining 150 psychiatric patients and 26 controls, we utilized “connectopic gradients” to capture the functional topographic organizations of striatal connectivity during resting-state scans conducted before and after stress induction. Connectivity gradients in rest and under stress were linked to different CA types and their frequency by Spearman correlation. Linear mixed models and moderation models were built to clarify the role of symptom strengths in these correlations. We found one type of CA—emotional neglect negatively predicted the post-stress-induction gradient shape, and stress reactive changes in the anterior-posterior orientation of the first-order striatal gradient. Moderation models revealed the observed correlations were selectively present in individuals with elevated comorbidity. Our results may provide new psychopathology-related biomarkers by tracking stress-induced changes in the general motivation systems. This demonstrates new perspectives in characterizing the striatal network and understanding its alterations in response to adverse childhood experiences.

## 1 Introduction

Childhood adversity (CA) refers to a wide range of negative impactful experiences during childhood (McLaughlin, 2018;McLaughlin et al., 2014). These experiences can include various forms of abuse, exposure to violence, emotional and physical neglect, as well as long-term poverty (McLaughlin et al., 2019;Moreno-López et al., 2020). CA heightens the risk for psychiatric disorders by altering brain-development trajectories during the highly adaptive periods of childhood (Teicher et al., 2016). One notable psychopathological consequence of CA is its impact on individual’s reactivity to acute stress later in life. Typically, after encountering stress, the reactivity of the autonomic nervous system and hypothalamic–pituitary–adrenocortical (HPA) axis contributes to effective coping (Russell & Lightman, 2019). However, individuals with a history CA often exhibit diminished cardiac and cortisol responses to psychosocial stress (Lovallo et al., 2012;Sijtsema et al., 2015). This maladaptive alteration is indicative of an increased likelihood of experiencing more depressive symptoms both currently and in the future (de Rooij et al., 2010;Phillips et al., 2011), and observed in several other psychiatric disorders besides depression, for example, disordered eating (Ginty et al., 2012), attention-deficit/hyperactivity disorder (ADHD;Pesonen et al., 2011), and substance use dependencies (Ginty et al., 2012). Recently, after integrating evidence from clinical and neuroscience research,Carroll et al. (2012,^2017^) proposed that in addition to changes in peripheral physiology, the hypothalamus and brainstem, blunted stress response may be also due to motivational dysregulation. That is, the decreased stress response and related health outcomes usually come together with impairments in motivation-dependent tasks, indicating a broader dysfunction in responding to challenges and mobilizing mental effort (Ginty, 2013). During exposures to stress, hypoactivation of motivation-related brain regions attenuates conscious engagement in appraising the present state and mobilizing resources. It will result in ineffective stress evaluation and strategic coping, thereby limiting the necessary adjustments for further physiological responses. Insufficient cortisol secretion and motivational coping after stress lead to long-term dysphoria, heightening the risks of depressive symptoms, substance abuse, and other maladaptive behaviors (al’Absi et al., 2021;Lovallo, 2013), therefore accounting for parts of the comorbidity related to CA and stress.

The striatum, together with its associated cortical regions (e.g., orbitofrontal cortex, medial prefrontal cortex (mPFC), anterior cingulate cortex), constitutes the essential parts of human reward and motivation system. The aforementioned perspective on motivational dysregulation (Carroll et al., 2017) highlights the potential involvement of the striatum and its cortical connectivity in stress-coping behaviors, particularly for elucidating the blunted stress response observed in individuals with a history of CA. Evidence showed that the experience of CA was associated with muted activation of the ventral striatum (Hanson et al., 2015) and striatum-related connectivity changes (e.g., striatum-mPFC connectivity (Dennison et al., 2019;Hanson et al., 2018)) during reward processing. Studies in resting-state functional connectivity also identified the CA-related alterations in the striatum-mPFC pathway, although the direction of these changes is inconsistent (Increase:Fareri et al., 2017; Decrease:Marshall et al., 2018). There is little research directly examining the relation between CA and striatal networks during stress response. One study found a negative correlation between CA severity and cortico-striatal activities in response to threat stimuli (Yang et al., 2015). Individuals experiencing long-term psychosocial adversity have been shown to exhibit dampened striatal dopaminergic function after stress induction (Bloomfield et al., 2019), indicating the linkage between CA and striatal dysfunctions during stress coping.

These results generally support the motivational account for blunted stress response in people with CA. However, there is a need for more direct evidence on how striatal connectivity varies under the combined impact of acute stress and aversive events experienced early in life. Additionally, most studies have focused on the activity of the striatum or specific connectivity pathways between the striatum and other brain regions. Employing methods that examine whole-brain connectivity can be highly insightful in clarifying the large-scale brain effects of the interaction between stress response and CA (Herzberg & Gunnar, 2020), providing a readout of the living human’s motivation system.

An emerging functional connectivity analysis technique, the “connectopic mapping,” could serve as an ideal tool to investigate this interaction in vivo and allow for investigation of whole-brain effects of the striatal system. This data-driven method was designed to detect several overlapping connectivity elements (gradients) within a pre-defined region-of-interest (ROI). Each connectivity gradient maps a varying topographic mode of connectivity changes within the ROI in relation to the rest of the brain (Haak et al., 2018). According to animal anatomical studies, projections from cortical regions are overlapping and topographically organized within the striatum (Haber et al., 2000,^2006^). Previous research has demonstrated that this organization in the striatum can be effectively captured by connectopic gradients (Marquand et al., 2017). Furthermore, recent studies showed that variations in striatal gradients mapped precisely onto the individual difference in a set of motivation-related tasks (e.g., delay discounting, sustained attention) and the psychological well-being (Marquand et al., 2017). Gradients could predict the comorbidity between psychiatric disorders (Mulders et al., 2022) and Parkinson’s disease severity as well (Oldehinkel et al., 2022), which is another disorder associated with reward processing and striatal dysfunction (Cools et al., 2022). Connectivity gradients of the striatum could, therefore, be an in-vivo readout of the functionality of reward and motivation processing. By estimating gradient maps in resting-state functional connectivity data after stress induction, we can assess the functional state of the motivational system during stress response and coping behaviors, and examine its individual variations related to CA.

In this study, we conducted an analysis of connectopic gradients using data from the “Measuring Integrated Novel Dimensions in Neurodevelopmental and Stress-related Mental Disorders” (MIND-Set) study (van Eijndhoven et al., 2022), to examine whether striatal connectivity gradients vary depending on CA experience, and how acute stress interacts with this pattern. As the high prevalence of comorbidity and limitations of symptom-based classification have been widely recognized, the idea of incorporating data across distinct diagnostic domains to identify common underpinnings and biological markers is advocated by the Research Domain Criteria (RDoC;Insel et al., 2010) and increasingly becoming a trend in recent research (Buckholtz & Meyer-Lindenberg, 2012;Guineau et al., 2023;Kist et al., 2023). Following this framework, the MIND-Set study comprises a naturalistic psychiatric patient sample and a non-psychiatric control sample, aiming to explore shared mechanisms and risk factors across neurodevelopmental disorders, stress-related disorders, and substance use disorders. Based on this rationale, we examined whether CA could play a role in the shared mechanisms, specifically concerning the striatal motivation system and stress response. Previous research has indicated diverse vulnerable regions underlying different forms of CA, characterized by different profiles of threat and deprivation (McLaughlin et al., 2014). Therefore, we investigated different CA types and their respective frequency. The literature summarized above has demonstrated that individuals with CA exhibit alterations in their striatal networks both before and after stress exposures. We hypothesized that these alterations would be reflected in differences in the spatial layouts of gradient maps. To be specific: 1.) participants with different types and frequencies of CA were expected to display different gradient organizations at the pre-stress baseline; 2.) after acute stress induction, we expected to observe distinct changes in gradient organization in individuals with different CA histories (in terms of types and frequency). If CA and acute stress, two critical concepts for mental health, are indeed reflected in connectivity gradients, these gradients will demonstrate sensitivity in detecting both individual characteristics and experimental manipulations. This indicates the future utility as biomarker for diagnosis, monitoring, and prediction of treatment response in mental health disorders.

## 2 Methods

### 2.1 Participants

The data used in this study are part of the MIND-Set study, conducted by the Department of Psychiatry of the Radboud University Medical Center and the Donders Institute in Nijmegen, the Netherlands. The MIND-Set study was approved by the Ethical Review Board of the Radboud UMC. All participants signed informed consent before participation. A more detailed description of the MIND-Set study is introduced in prior work (van Eijndhoven et al., 2022;https://scholar.google.nl/citations?user=GBcK84EAAAAJ&hl=nl).

The aim of the present study was to investigate the relations between CA and striatal connectivity, therefore all the participants from the MIND-Set cohort who have experienced CA (measured by NEMESIS questionnaire, details in 2.2 Procedure) and have imaging data available (*n*= 176; 94 males; age 38.7 ± 14.2 years) were included. In this sample, 150 participants diagnosed with one or more psychiatric disorders (79 males; age 38.9 ± 13.6 years; mood disorder = 131, anxiety disorder = 51, ADHD = 58, autism spectrum disorder (ASD) = 55, addiction = 40), and 26 individuals without a current or past psychiatric disorder (15 males; age 37.6 ± 17.7 years). The diagnostic procedure is described in detail byvan Eijndhoven et al. (2022). During their participation in the study, 126 participants in this sample were taking one or more medications.

### 2.2 Procedure

**Fig. 1.**
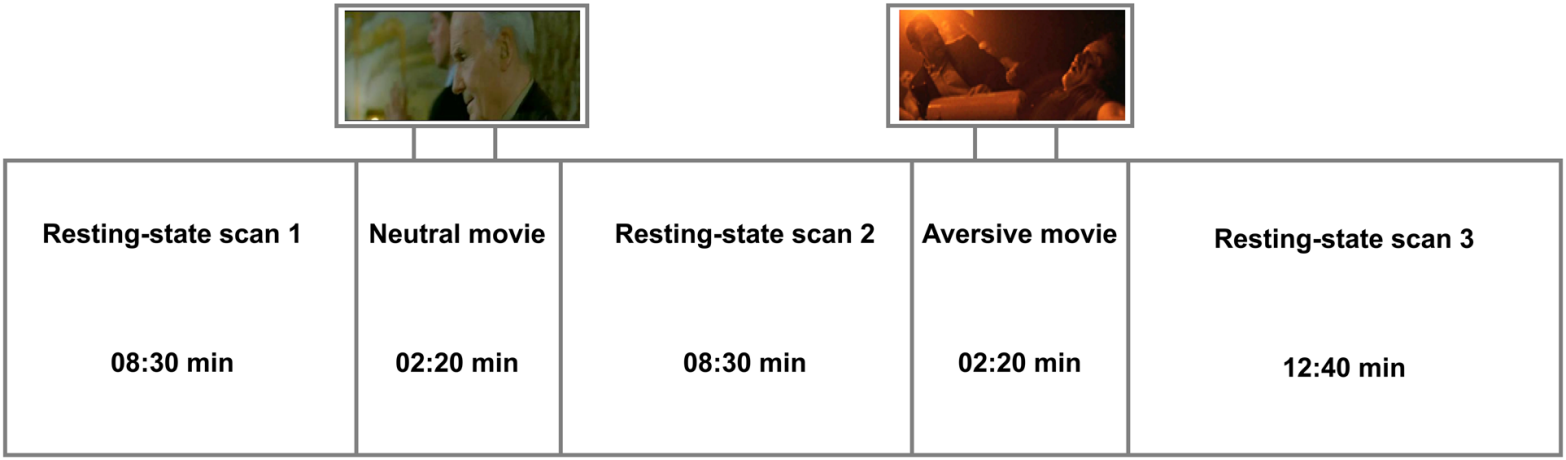
Resting-state scan procedure (adapted fromvan Oort et al., 2020).

In this study, we utilized neuroimaging data from resting-state scans. There are three resting-state scans in total (Fig. 1). Resting-state scan 1 (8.5 min) was followed by a neutral movie clip (2.3 min; control condition), in turn followed by resting-state scan 2 (pre-stress rs, 8.5 min). Lastly, to induce psychological stress, participants watched a highly aversive movie clip (2.3 min;Hermans et al., 2011;Qin et al., 2009) after which resting-state scan 3 (post-induction rs, 12.6 min) was acquired. Because the main focus of this study is on stress reactivity, performed analyses were based on pre-stress and post-induction rs.

CA was measured by the Netherlands Mental Health Survey and Incidence Study (NEMESIS;Bergman et al., 2020) questionnaire. Participants indicated whether and how frequently they experienced emotional neglect, psychological abuse, physical abuse, and sexual abuse, each of which was categorized into three degrees (0: absent, 1: once or sometimes, 2: regular, often and very often). The overall CA index was computed by summing up the four subscales, ranging from 0 to 8 (Hovens et al., 2010).

### 2.3 Analysis in stress indicators

To examine whether the aversive movie effectively induced participants’ stress state, the paired-sample T test (*p*< 0.05, two tailed) was utilized to compare the subjective stress rating and the heart rate (beat per minute, BPM) after watching the neutral movie and the aversive movie. To further examine how CA relates to these stress indicators, we did the spearman correlation analyses (*p*< 0.05) between different types of CA and the stress-induced changes in subjective stress ratings and BPM.

### 2.4 fMRI data analysis

The fMRI acquisition parameters and preprocessing pipeline were reported in previous work (van Eijndhoven et al., 2022;van Oort et al., 2020). In brief, performed by FSL 5.0.11 (FMRIB, Oxford, UK), the preprocessing steps consist of brain extraction, motion correction, bias field correction, high-pass temporal filtering (cut-off of 100 s), spatial smoothing by a 4 mm FWHM Gaussian kernel, boundary-based registration to T1, and nonlinear registration to standard space (MNI152). ICA-based Advanced Removal of Motion Artefacts (ICA-AROMA) was applied to remove motion-related artifacts from the data.

We applied ConGrads to the denoised resting-state data, to find varying topographic modes of connectivity changes within the striatum in relation to all the other regions of the brain (Haak et al., 2018). For our ROIs of left and right striatum, we produced masks from the Harvard-Oxford atlas using the thresholding of 25% probability (Oldehinkel et al., 2022). ConGrads estimated a similarity matrix based on functional connectivity between each striatal voxel and the rest brain, and utilized a manifold learning algorithm to derive topographical gradient maps from the similarity matrix: similar values in the gradient maps indicate a similar connectivity pattern. Then, the trend surface model (TSM) was fitted to statistically represent the spatial organization of gradient maps (Haak et al., 2018;Marquand et al., 2017). We run ConGrads separately for the pre-stress and post-induction rs (each 500 vol), and the individual connectopic maps were obtained for each participant at respective resting-states on both sides of the striatum. All connectopic maps were checked visually. We focused our analysis on the first-order gradient as it has been shown to be associated with a set of goal-directed behaviors, as well as psychological well-being (Marquand et al., 2017). A template derived from previous publications (Oldehinkel et al., 2022) and calculated using data from the Human Connectome Project (HCP) was utilized for the group gradient. To validate correct selection of first-order gradients, participants were excluded if the spatial correlations between their gradients and group level first-order gradients were lower than 0.50. This criterion ensured that only participants whose gradient maps generally aligned with the group template were included in the subsequent analyses.

### 2.5 Linking striatal gradients with CA and acute stress

ConGrads provides trend surface modeling coefficients summarizing the spatial topology of each gradient in the X, Y, and Z axes of the standard MNI152 coordinate space. Trend surface modeling aims to estimate the value of a property (P_i_) at any given location within the space based on the coordinates (X_i_, Yi, Z_i_) of that location through regression functions, and therefore provide a set of coefficients from the regression function to serve as low dimensional representations of the spatial trend (Gelfand et al., 2010). Generally, the similarity in the structure of these coefficients suggests similarities in spatial layouts. In line with prior research applying striatal gradients to the MIND-Set data (Mulders et al., 2022), a trend surface regression model with nine coefficients (3 parameters along 3 axes) was determined to be the best fit, thus it was implemented in the analysis of the acquired first-order gradients. In order to investigate the stress effect on spatial features of the striatal gradient, we subtracted the nine parameters of the gradient at pre-stress resting state from the respective parameters at post-induction resting state, for each participant.

Considering the skewed distribution of the overall CA index and the subscale scores, Spearman correlation coefficients (*p*< 0.05, FDR corrected) were calculated to identify the relations between CA (overall index and three subscale scores: emotional neglect, psychological abuse, physical abuse) and the nine coefficients of the first-order gradient (separately for the two resting-states, and the stress induced changes). The subscale of sexual abuse was discarded due to its extremely skewed distribution. The correlation analysis was done by the R package “bcdstats” (github.com/bcdudek/bcdstats) based on R version 4.2.0.

### 2.6 Dependence with depressive severity and comorbidity

The motivational dysfunction after acute stress may, indeed, be associated with depressive symptoms and the comorbidity across psychiatric disorders, and disentangling the relationships between CA, depressive severity, and comorbidity has proven challenging (Abercrombie et al., 2018;Vrijsen et al., 2017). Therefore, it is necessary to examine how the observed significant correlations in our study are related to these symptom strength factors. It is possible that the correlations between CA and striatal gradients might be in fact dominated by depressive symptom severity and levels of comorbidity, or they play specific roles (e.g., moderating) in the relation of interest. To explore these possibilities, linear mixed models were built by taking CA and these symptom indicators as factors, using R package “lmerTest” (Kuznetsova et al., 2017). Here, the depressive severity was measured by Inventory of Depressive Symptomatology–Self Rating (IDS-SR;Rush et al., 2000), and the comorbidity was calculated by summing up the number of psychiatric disorders (including mood disorder, anxiety disorder, ADHD, ASD, and addiction) diagnosed for each participant.

Specifically, based on the results from Spearman correlation analysis, we built linear mixed models on the Y cubic parameter of the left striatum (the dependent factor), taking emotional neglect frequency, resting state (pre-stress rs, post-induction rs), depressive severity, or comorbidity as fixed factors, and subjects as the random factor. We also ran similar linear mixed models on the stress-induced change of the Y cubic parameter, with emotional neglect frequency, sides of the striatum (left, right), the depressive severity, or comorbidity as fixed factors, and subjects as the random factor.

## 3 Results

### 3.1 Stress indicators

Compared to watching the neutral movie, the stress induction with aversive movie significant improved participants’ subjective stress ratings (*t*(170) = 11.136,*p*< 0.001, Cohen’s d = 0.851) and BPM (*t*(172) = 3.255,*p*= 0.001, Cohen’s d = 0.247). Furthermore, we found a marginal significantly negative correlation between emotional neglect frequency and the BPM change (*r*_s_= -0.142,*p*= 0.062; seeSupplementary Table S1), showing the evidence that CA was related to diminished cardiac responses to stress.

### 3.2 Striatal connectivity gradients in MIND-Set Data

Our results showed similar striatal gradients compared with previous studies (Marquand et al., 2017;Mulders et al., 2022;Oldehinkel et al., 2022), which confirmed the reliability of striatal gradients across different populations and scans. The dominant gradient (zeroth-order) essentially follows the structural boundaries of the caudate, putamen, and nucleus accumbens, while the first-order gradient tends to show changes following the coordinate space (e.g., medial–lateral, dorsal–ventral, and anterior–posterior; seeFig. 2). For the first-order gradient, there was a high proportion of participants whose spatial correlation with the HCP group gradient was higher than 0.50 (left striatum pre-stress: 84.09%; right striatum pre-stress: 83.52%; left striatum post-induction: 86.36%; right striatum post-induction: 81.81%).

**Fig. 2.**
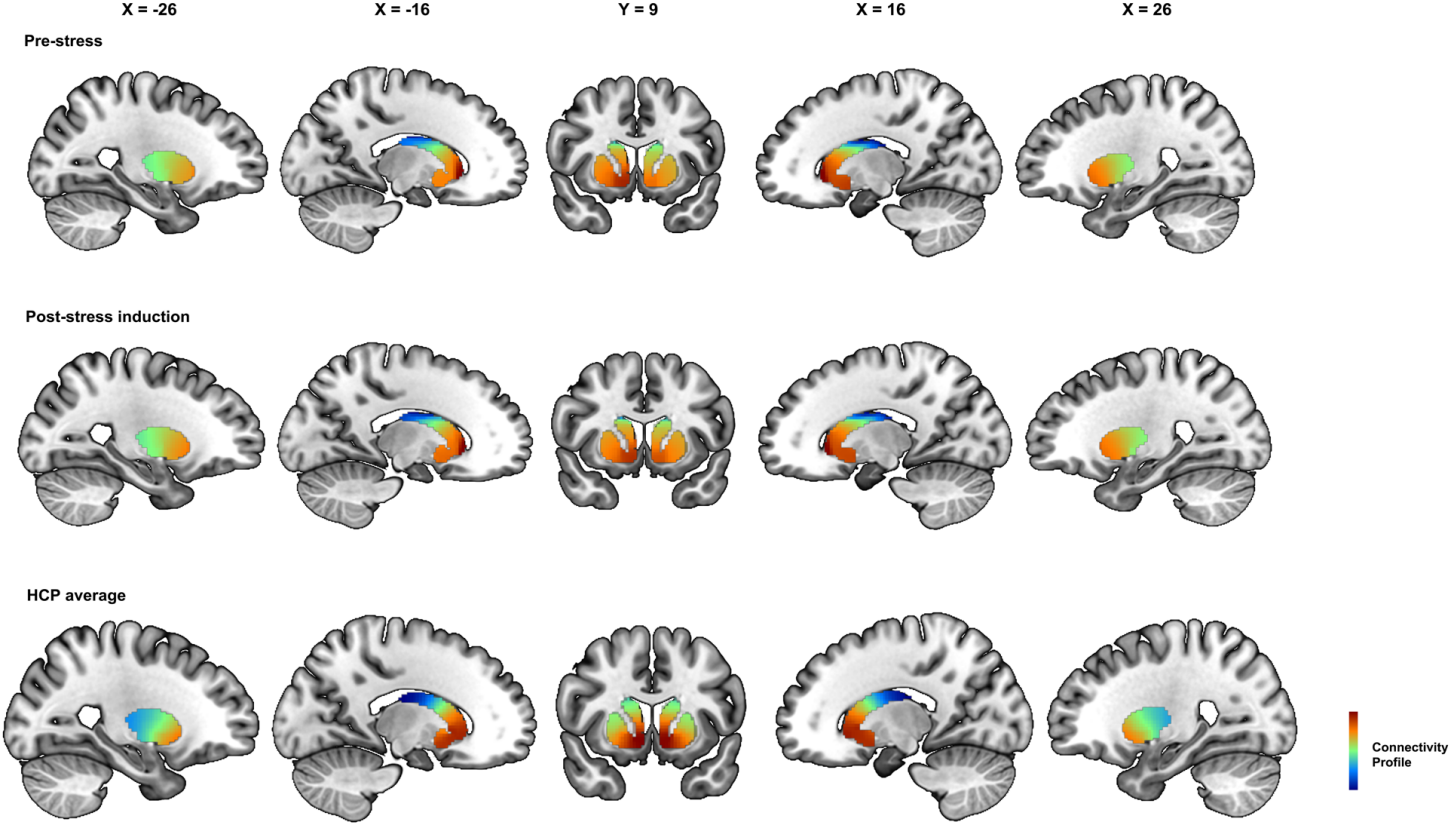
The group average of the first-order gradients before and after stress-induction, compared with HCP dataset (Oldehinkel et al., 2022).

### 3.3 Correlation between different types of CA and striatal gradients

The Spearman correlation analyses between CA and the TSM coefficients from the first-order gradient, conducted separately for the pre-stress and post-induction resting state, showed a significant negative correlation only between emotional neglect frequency and the Y cubic parameter (the cubic parameter representing anterior-posterior organizations) of the first-order gradient in the left striatum at post-induction rs (*r_s_*= -0.190,*p*_fdr_= 0.042).

**Fig. 3.**
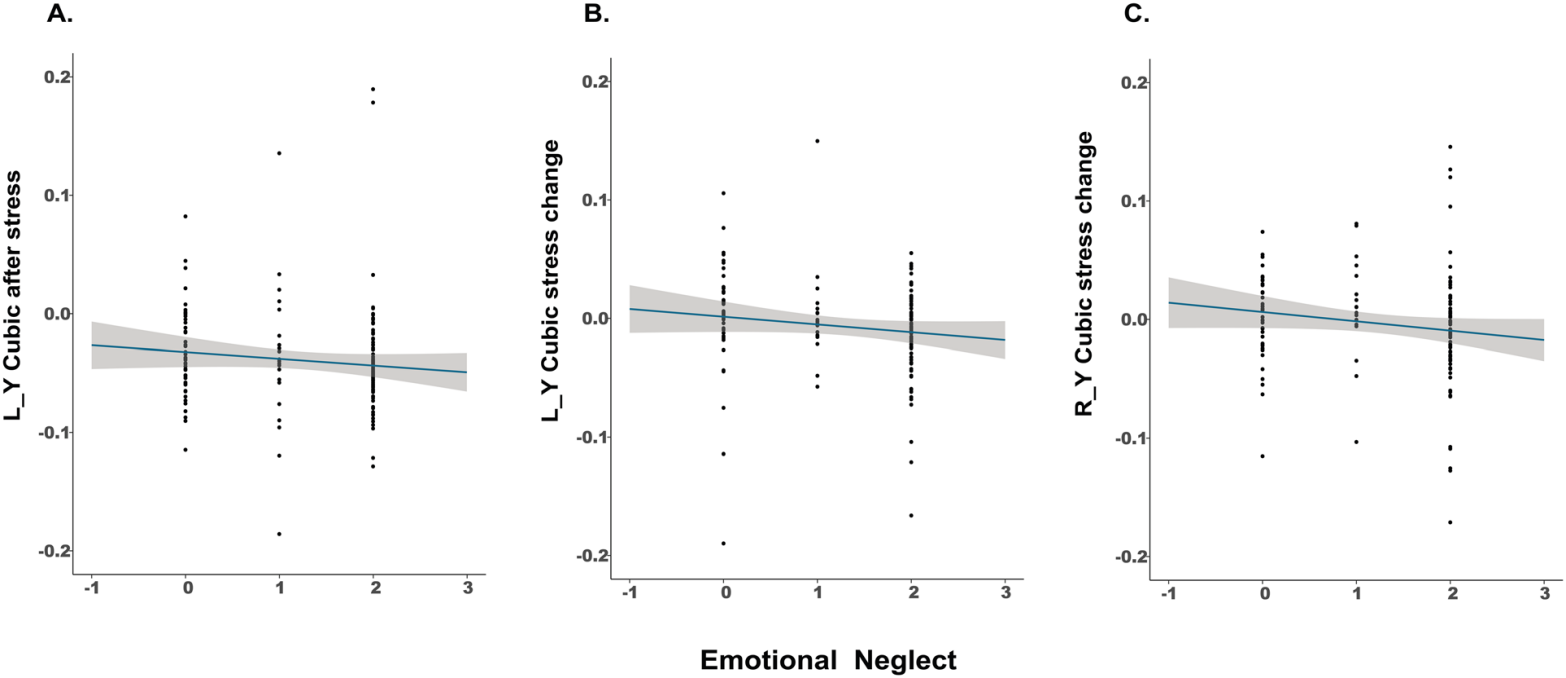
Emotional neglect predicts striatal connectivity gradient changes in response to stress, shown by correlations between emotional neglect and Y cubic parameter on the left striatum at post-induction rs (A), Y cubic parameter changes induced by acute stress on the left (B) and right striatum (C).

For the stress-induced connectivity change, we found significant negative correlations between emotional neglect frequency and the Y cubic parameter change on both sides of the striatum (left:*r_s_*= -0.210,*p*_fdr_= 0.033; right:*r_s_*= -0.230,*p*_fdr_= 0.014;Fig. 3). There were no other significant correlations (*p*s > 0.10). The full correlation lists are shown inSupplementary Table S2.

To assess the change in correlations from pre-stress rs to post-induction rs, we utilized the Pearson and Filon’s Z test (Pearson & Filon, 1898) by the R package “cocor” (Diedenhofen & Musch, 2015), comparing the correlation between emotional neglect frequency and the Y cubic parameter from the two resting states. The results showed for both sides of the striatum, the correlations between emotional neglect frequency and the Y cubic parameter were significantly different between the two resting-states (left:*z*= 2.789,*p*= 0.005; right:*z*= 2.348,*p*= 0.019).

We also tested the above correlations controlling for gender, age, and medication use (i.e., with/without medication). The negative correlations between emotional neglect frequency and the Y cubic parameter change on both sides of the striatum remained significant (left:*r_s_*= -0.220,*p*_fdr_= 0.021; right:*r_s_*= -0.200,*p*_fdr_= 0.038). The correlation between emotional neglect frequency and the Y cubic parameter on the left striatum at post-induction rs was marginally significant (*r_s_*= -0.170,*p*_fdr_= 0.088). The full overview of correlations is presented inSupplementary Table S3.

Because the significant correlations we found all included the Y cubic parameter and emotional neglect frequency, we examined whether the correlations with emotional neglect were statistically different from those for the other types of CA, using Pearson and Filon’s Z test. This analysis found that the correlation between emotional neglect frequency and the left Y cubic parameter was significantly different from physical abuse frequency and the respective parameter (*z*= -1.910,*p*= 0.028); the correlation between emotional neglect frequency and the right Y cubic parameter change was significantly different from psychological abuse frequency (*z*= -2,923,*p*= 0.002) and physical abuse frequency (*z*= -1.925,*p*= 0.027; seeSupplementary Table S4). Overall, this indicates potentially specific linkage between emotional neglect and the examined gradients.

Moreover, to better interpret the observed correlations, for each hemisphere of the striatum, we ran repeated-measures ANOVA for the Y cubic parameter, taking emotional neglect frequency (low, high) and resting state (pre-stress rs, post-induction rs) as independent factors. Here, we classified participants who scored 0 and 1 as the low-frequency group, and participants who scored 2 as the high-frequency group, to get a relatively balanced group size (n_0_= 59; n_1_= 23; n_2_= 94). The analysis in the right striatum revealed a significant interaction between emotional neglect frequency and resting state (*F*(1, 129) = 4.102,*p*= 0.045). It was driven by the decrease of Y cubic parameter from pre-stress rs to post-induction rs in high-frequency group (*p*= 0.049), while the low-frequency group did not show this change (*p*= 0.353). Similar results were shown in the left hemisphere as well, but did not reach significance (*F*(1, 134) = 3.172,*p*= 0.077).

**Fig. 4.**
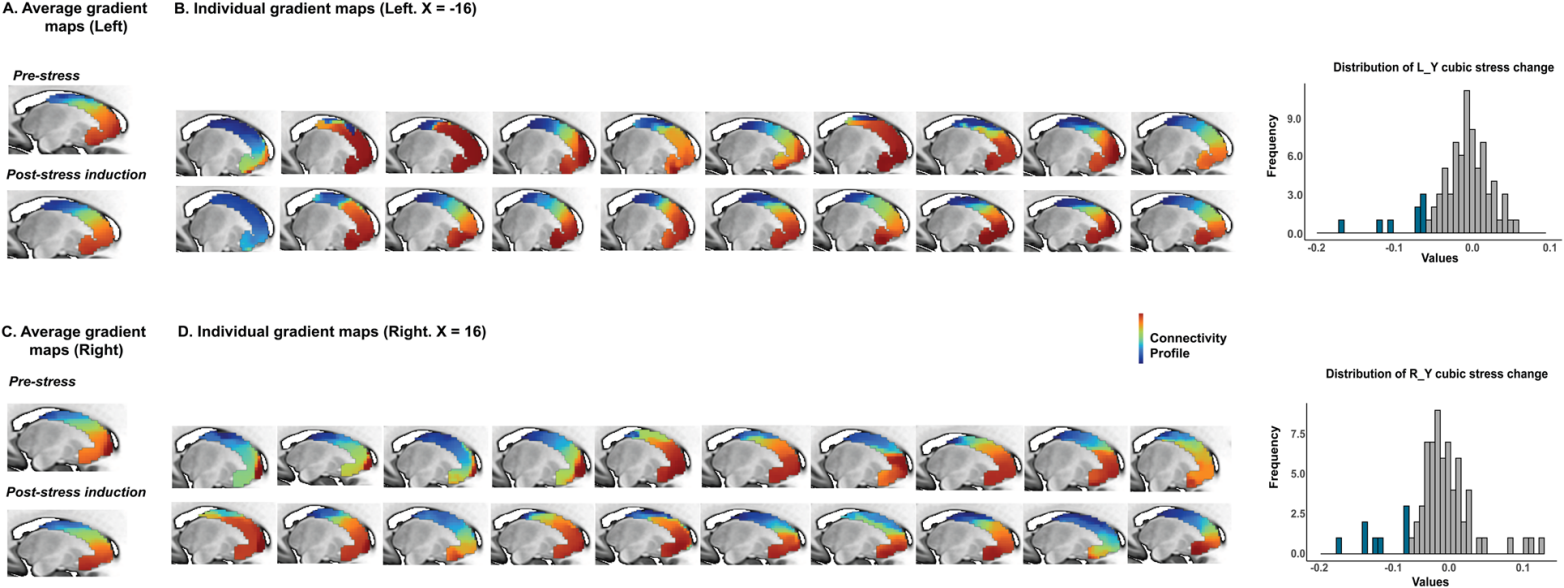
The pre-stress and post-induction gradient maps for frequently emotional neglected participants. (A) and (C) depict the average maps derived from 10 participants in the high-frequency group who exhibited the largest decrease in the Y cubic parameter at the left and right striatum (shown as blue bins in the distribution histograms), respectively. (B) and (D) display the individual maps of these participants. In comparison to the pre-stress state, their gradient maps tended to exhibit clearer and more gradual transitions in spatial organizations during the post-induction state.

These results indicated the correlations we observed above are related to the stress-induced decrease in the high frequency of emotional neglect group. We next attempted to visualize this effect in the individual striatal gradient maps. We visually inspected all the individual gradients of the high-frequency group. Then, for each side of the striatum, we produced the average gradient maps from 10 participants of the high-frequency group who showed the largest stress-induced decrease in their Y cubic parameter, and compared the average maps at pre-stress and post-induction rs (Fig. 4). Combining the information from individual and average maps, we made the inference that the first-order gradients of frequently neglected people tend to show a clearer gradual transition from pre-stress rs to post-induction rs, especially in the location of caudates.

### 3.4 The role of psychiatric comorbidity and depressive symptoms

**Fig. 5.**
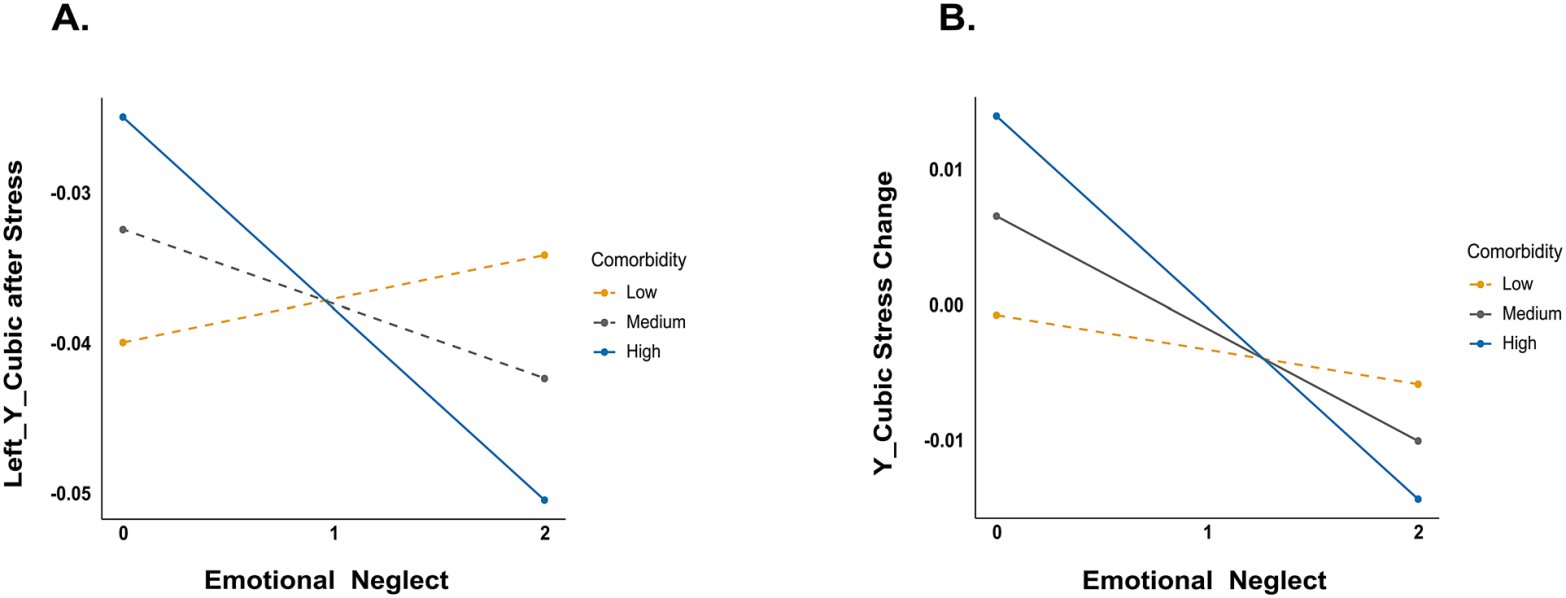
The illustration for moderation models. The predictive effects of emotional neglect on the Y cubic parameter of the left striatum at post-stress induction (A) and the stress-induced changes of this parameter (B) vary based on levels of comorbidity. The dashed lines represent insignificant predictions, and solid lines represent significant predictions.

As both stress reactivity and childhood adversity are associated to psychiatric symptoms and depression in particular and we are investigating a clinical sample, we tested the potential role of depressive severity and comorbidity on the correlation between emotional neglect frequency and the Y cubic parameter on the left striatum at the post-induction rs. For this purpose, linear mixed models were built on the Y cubic parameter of the left striatum as the dependent variable, taking emotional neglect frequency, resting state (the repeated measure: pre-stress rs, post-induction rs), depressive severity, or comorbidity as fixed factors, and subjects as the random factor. The summary of these models is shown inSupplementary Table S5. The model with the depressive severity found no significant associations (all*p*s > 0.36), indicating the effect of emotional neglect frequency may prove difficult to disentangle from participants’ depressive state (e.g., neither of them could explain the variance in gradients independently). In contrast, the model with comorbidity found a significant interaction between resting state, emotional neglect frequency, and comorbidity (*F*(1, 129.94) = 5.172,*p*= 0.025; seeSupplementary Table S6). In order to further elucidate the nature of the relations between emotional neglect frequency and the Y cubic parameter in interaction with different comorbidity levels, we tested whether levels of comorbidity moderated the relation between emotional neglect frequency and the Y cubic parameter separately for pre-stress rs and post-induction rs. In these models, the Y cubic parameter was the dependent variable, emotional neglect frequency the independent variable, and comorbidity the moderator. These moderation models were tested using PROCESS v2.16 for SPSS (Hayes, 2018), with the bootstrap samples of 5,000 and a confidence level of 95%. The model for post-induction resting state found a significant interaction between emotional neglect frequency and comorbidity (*F*(1, 147) = 4.831,*p*= 0.030; seeSupplementary Table S7). The conditional effect analysis showed that at low and medium levels of comorbidity, the prediction from emotional neglect frequency was not significant (*p*s > 0.24); while for individuals with high comorbidity, emotional neglect frequency negatively predicted the Y cubic parameter (*t*= -2.212,*p*= 0.029;Fig. 5A). It is noteworthy that these results should be inspected with caution as the test for the entire model was not significant (*F*(3, 147) = 2.122,*p*= 0.100). For the pre-stress-rs model, neither the whole model nor the interaction was significant (*p*s > 0.50).

To test whether this difference in models reflects a significant stress-related change, we also ran similar linear mixed models on the stress-induced change of the Y cubic parameter (the dependent variable). We included emotional neglect frequency, sides of the striatum (the repeated measure: left, right), the depressive severity, or comorbidity as fixed factors, and subjects as the random factor. Similarly, the model with the depressive severity found no significant associations (all*p*s > 0.09), whereas the model with comorbidity found a significant interaction between emotional neglect frequency and comorbidity (*F*(1, 127.67) = 3.985,*p*= 0.048). The successive moderating effect analysis further elucidated this interaction (*F*(1, 261) = 4.546,*p*= 0.034): for participants with low levels of comorbidity, the predicting effect of emotional neglect frequency was not significant (*p*= 0.52); while for individuals with medium and high comorbidity, emotional neglect frequency negatively predicted the Y cubic parameter change (medium:*t*= -2.637,*p*= 0.009; high:*t*= -3.191,*p*= 0.002;Fig. 5B). The whole moderating model was also significant (*F*(3, 261) = 3.457,*p*= 0.017).

The results above show the same pattern after controlling for gender, age, and medication use (seeSupplementary Tables S8 and S9).

## 4 Discussion

As far as we know, this study represents the first investigation focusing on striatal connectivity gradients to investigate the inter-individual relations to CA and psychiatric comorbidity, as well as intra-individual modulation of these gradients by acute stress. Through applying connectopic mappings to the psychiatric sample of MIND-Set, we found for the first-order striatal gradient: 1.) participants with different CA histories did not exhibit significantly diverse gradients at the pre-stress baseline, as the connectopic maps could not be predicted by any types of CA; 2.) after the stress-induction, this gradient map became associated with one type of CA, specifically emotional neglect. Additionally, emotional neglect frequency negatively predicted the stress reactive change in this connectivity mode; 3.) the observed correlations between emotional neglect and striatal gradients only existed in individuals with elevated comorbidity. By highlighting the joint impact of CA and acute stress, our findings contribute to the potential utility of striatal connectivity gradients in understanding and, potentially, diagnosing and treating psychiatric disorders in the future.

Previous studies have reported CA to be associated with a blunted stress response in the HPA axis and cardiac functions (Carpenter et al., 2007;Elzinga et al., 2008;Sijtsema et al., 2015). The recent model proposed this blunted response might be related to the motivation function (Carroll et al., 2012,^2017^): people with CA are less motivated to engage in coping with stress, due to their functional changes in the striatum and related cortical regions (e.g., mPFC) shaped by CA. Potentially in line with this perspective, we found that emotional neglect was negatively correlated with the stress-induced change of the anterior-posterior organization of the first-order striatal gradients on both sides of the striatum. The first-order striatal gradient has been shown to be associated with goal-directed behaviors (e.g., delay discounting, relational processing, social cognition, sustained attention) and psychological well-being (Marquand et al., 2017), which largely rely on the corticostriatal circuitry, especially the striatum-PFC interaction (Balleine et al., 2007;Barch et al., 2013). Therefore, to some extent, our results provide new evidence for the motivation account of the stress response in CA at the brain connectivity level, as we did observe the motivation-related gradient change under the interaction of CA and acute stress. Moreover, the study byHanson et al. (2018)identified the striatum-PFC connectivity to be a biological link between stress exposure and internalizing depressive symptomatology in adolescents with childhood maltreatment. Taking together, we could infer the corticostriatal connectivity mode, reflected by the first-order striatal gradient, possibly contributing to the neural substrate for high-order cognitive functions (e.g., motivations to cope with stress), is vulnerable to early-life adversity and further linked to psychopathology of mental disorders, for example, depression; and functional connectivity gradients might provide an individual read-out of the resulting malfunction.

Although our analyses included three types of CA and the overall CA index in an attempt to capture the variance in frequency that encompasses all CA types, we found correlations between the first-order gradient and the frequency of one specific CA—emotional neglect, which also proved different from the respective correlations with other CA types. Emotional neglect describes maltreatment behaviors failing to meet children’s emotion needs and provide enough nurturance and affective support (Stoltenborgh et al., 2013), which lead to a lack of sufficient cognitive and social input for the brain during development. Studies support the notion of separating different types of adversity: while threatening violent behavior (e.g., abuse) affects limbic regions (e.g., hippocampus and amygdala) and their regulations from the cortex, forms of neglect affect cortical areas such as PFC, superior, parietal, and temporal cortex, which are involved in complex cognitive and social tasks (McLaughlin et al., 2014;Wu et al., 2023). Additionally, studies among healthy adults have observed structural changes in emotional regulation and motivation regions specifically related to neglect (Everaerd et al., 2015;Tendolkar et al., 2018). Our results identified relations between emotional neglect and the connectivity gradient associated with goal-directed behaviors, which are aligned with the previous viewpoint about neglect, but also link it with the functional coupling of the striatum. It could be inferred that scarce emotional inputs during childhood would limit the knowledge, motivation, and ability to process and regulate emotions, especially under the situation of dealing with acute stress. It is noteworthy that our results do not necessarily imply that other types of CA and the overall CA index have no relations with striatal connectivity modes, as here we only examined the first-order gradients and the frequency of each CA type. Future research can explore further whether CA works as an all-or-none manner, and if the level of severity matters.

From the aspect of gradients, our results consistently pointed to the cubic anterior-posterior organization of the first-order gradient: both the stress-induced connectivity change and the post-stress-induction status were related to emotional neglect frequency. The cubic parameter specifically represents the quadratic coefficient for the derivative of cubic functions. It determines the distribution of changing rates along the anterior-posterior direction within the space. These results indicated the spatial changing pattern of connectopic modes along the Y direction was related to CA and stress. Subsequent analyses demonstrated that this association was mainly driven by changes in high frequency of neglect group. Stress induction might “reset” the anterior-posterior organization of the striatal connectivity gradient for these people, as reflected by the spatial changing pattern. In an attempt to visualize and thereby better understand the re-organization, we visually checked all the gradient maps, and produced the average maps with participants showing largest changes in the direction of observed correlations, assuming these extreme cases represent the possible pattern more obviously. By comparing gradient maps for the two resting-state scans, we realized that the topographic shape of the high-frequency group tended to show a more pronounced gradual change pattern at post-stress rs, which is more similar to group gradients in the healthy sample (e.g., HCP dataset). This potential pattern can be considered together with the motivation model above: more structured gradients reflect a normal state of motivation network, which might indicate that the motivation system of these individuals functions more normally under elevated stress. Importantly, the results did not show any difference related to emotional neglect at the pre-stress rs, so we could not suppose an impaired baseline striatal connectivity mode for the high-frequency group. What we found only captured the stress-induced change specifically for this group.

CA was shown to be consistently related to depression (Abercrombie et al., 2018;Beck & Bredemeier, 2016) and psychiatric comorbidity (Vrijsen et al., 2017). Our linear mixed models with both depressive severity and emotional neglect frequency as fixed factors found that when controlling the effect of each other, neither depression nor emotional neglect could explain the significant variance of the anterior-posterior organization parameter and its stress-induced change independently. Due to the relatively high correlation between emotional neglect frequency and depressive symptom levels (*r_s_*= 0.292,*p*< 0.001) in our sample, we could not further explore the role of the depressive severity here. Future studies that include a broader and more independent range of variance in the two factors (e.g., individuals with high emotional neglect frequency and low depression, or low emotional neglect frequency and high depression) are necessary. In contrast, comorbidity seems to interact with emotional neglect and influence the striatal gradients: for both post-induction value and stress-induced gradient change, emotional neglect only showcases significant effects in individuals with elevated comorbidity, but not individuals with fewer psychiatric disorders. This finding highlights the psychopathological link between CA, stress, and striatal dysfunction. In this sense, the cubic anterior-posterior organization (Y cubic parameter) could be used as a new biomarker for the symptomatology of people with a frequent neglected history, by tracking stress-related brain changes in the general motivation and high-order cognition systems. The targeted diagnostics and treatment could be developed based on the findings.

In addition to these implications, we acknowledge certain limitations in our study. Firstly, to reduce participant drop-out and enhance data collection efficiency in the large database, stress induction via aversive movie watching was administered in the same session as the control task (neutral movie watching). To prevent the carry-over of stress states, stress induction was conducted after the control task, potentially introducing order effects. Future designs separating the two conditions into different sessions would facilitate a more refined examination of the stress effect. Secondly, the observed correlations fall within the small to medium range of effect size (Cohen, 1988;Funder & Ozer, 2019;Gignac & Szodorai, 2016). It is noteworthy that these correlations were consistently observed across the bilateral striatum, encompassing both the post-stress state and stress-induced changes in the same parameter, indicating a stable predictive pattern. However, a more balanced distribution in CA might be beneficial to validate the results and those analyses might potentially yield a larger effect. Lastly, the Y cubic parameter indeed demonstrates the ability to capture the interacting influence of stress and CA on striatal networks, while additional methods beyond visual inspection could aid in further interpretations, such as how to map differences in TSM parameters with more targeted biological changes.

In conclusion, our study showed that the frequency of emotional neglect predicted alterations in the striatal functional connectopic gradients following acute stress induction, with this association being selectively present in individuals with elevated comorbidity of psychiatric disorders. These findings may contribute to future explorations in the psychopathology of childhood adversity and the utility of functional connectivity gradients for the striatum in clinical applications.

## Supplementary Material

Supplementary Material

## Data Availability

The data and codes included in this study are stored in the institutional repository of the Donders Institute for Brain, Cognition and Behavior, and available upon reasonable request.
