# Supplementary material for: Childhood adversity predicts striatal functional connectivity gradient changes after acute stress
Source: Imaging Neurosci (Camb). 2024 Aug 19;2:imag-2-00269. doi: 10.1162/imag_a_00269 (PMC12290603; doi:10.1162/imag_a_00269)
Supplement: Supplementary Material [file imag_a_00269-supp.pdf]

Supplementary Material

Table S1. Spearman correlations between childhood adversity and stress-response indicators

		Emotional Neglect	Psychological Abuse	Physical Abuse	Childhood Trauma
**Δ Stress rating**	r_s	0.103	0.037	-0.102	0.075
	p	0.177	0.627	0.180	0.327
**Δ BPM**	r_s	-0.142	-0.018	0.023	-0.069
	p	0.062	0.812	0.767	0.366
N = 173					

Table S2. Spearman correlations between CA and the first-order gradient parameters

		Emotional Neglect	Psychological Abuse	Physical Abuse	Childhood Trauma
**rs2_Left_X Linear**	r_s	0.040	0.020	0.140	0.050
	p_{fdr}	0.778	0.861	0.190	0.771
**rs2_Left_Y Linear**	r_s	0.030	-0.040	-0.100	-0.010
	p_{fdr}	0.828	0.778	0.396	0.958
**rs2_Left_Z Linear**	r_s	0.030	-0.030	-0.070	0.000
	p_{fdr}	0.834	0.834	0.648	0.961
**rs2_Left_X quadratic**	r_s	0.100	0.080	-0.050	0.120
	p_{fdr}	0.388	0.573	0.778	0.273
**rs2_Left_Y quadratic**	r_s	0.030	0.030	0.010	0.010
	p_{fdr}	0.834	0.828	0.961	0.953
**rs2_Left_Z quadratic**	r_s	-0.100	-0.040	0.000	-0.110
	p_{fdr}	0.375	0.778	0.961	0.333
**rs2_Left_X cubic**	r_s	-0.040	-0.020	-0.120	-0.050
	p_{fdr}	0.778	0.861	0.300	0.771
**rs2_Left_Y cubic**	r_s	0.030	-0.060	0.030	-0.040
	p_{fdr}	0.834	0.657	0.834	0.778
**rs2_Left_Z cubic**	r_s	0.000	-0.080	0.030	-0.080
	p_{fdr}	0.961	0.533	0.834	0.573
N = 148					
**rs3_Left_X Linear**	r_s	-0.010	0.050	0.050	0.060
	p_{fdr}	0.981	0.618	0.625	0.595
**rs3_Left_Y Linear**	r_s	0.110	-0.080	-0.110	0.000
	p_{fdr}	0.282	0.440	0.284	0.986
**rs3_Left_Z Linear**	r_s	0.020	0.040	0.130	0.130
	p_{fdr}	0.846	0.677	0.219	0.224
**rs3_Left_X quadratic**	r_s	0.090	0.050	0.130	0.150
	p_{fdr}	0.407	0.643	0.219	0.134
**rs3_Left_Y quadratic**	r_s	-0.070	0.020	0.030	-0.030

	p_{fdr}	0.481	0.867	0.761	0.815
**rs3_Left_Z quadratic**	r_s	-0.090	-0.040	-0.070	-0.130
	p_{fdr}	0.398	0.681	0.481	0.223
**rs3_Left_X cubic**	r_s	0.010	-0.100	-0.070	-0.110
	p_{fdr}	0.981	0.343	0.491	0.317
**rs3_Left_Y cubic**	r_s	-0.190*	-0.050	0.000	-0.120
	p_{fdr}	0.042	0.618	0.987	0.243
**rs3_Left_Z cubic**	r_s	0.060	-0.100	-0.130	-0.080
	p_{fdr}	0.583	0.374	0.225	0.440
N = 152					
**ΔLeft_X Linear**	r_s	-0.020	0.000	-0.030	0.020
	p_{fdr}	0.933	0.994	0.886	0.918
**ΔLeft_Y Linear**	r_s	0.110	0.010	-0.020	0.030
	p_{fdr}	0.376	0.970	0.914	0.886
**ΔLeft_Z Linear2**	r_s	0.070	0.110	0.070	0.150
	p_{fdr}	0.625	0.325	0.634	0.166
**ΔLeft_X quadratic**	r_s	0.030	-0.020	0.010	0.020
	p_{fdr}	0.911	0.914	0.970	0.914
**ΔLeft_Y quadratic**	r_s	-0.150	-0.080	0.010	-0.100
	p_{fdr}	0.166	0.600	0.970	0.403
**ΔLeft_Z quadratic**	r_s	0.030	0.080	-0.060	0.020
	p_{fdr}	0.886	0.532	0.735	0.918
**ΔLeft_X cubic**	r_s	0.000	-0.010	0.030	-0.050
	p_{fdr}	0.970	0.949	0.895	0.816
**ΔLeft_Y cubic**	r_s	-0.210*	-0.070	-0.060	-0.130
	p_{fdr}	0.033	0.644	0.729	0.226
**ΔLeft_Z cubic**	r_s	-0.010	-0.040	-0.050	-0.030
	p_{fdr}	0.970	0.868	0.776	0.895
N = 136					
**rs2_Right_X Linear**	r_s	-0.010	0.020	-0.030	0.000
	p_{fdr}	0.978	0.913	0.913	0.978
**rs2_Right_Y Linear**	r_s	-0.050	0.000	-0.070	-0.010
	p_{fdr}	0.747	0.978	0.601	0.978
**rs2_Right_Z Linear**	r_s	0.030	0.000	0.160	0.020
	p_{fdr}	0.913	0.978	0.102	0.913
**rs2_Right_X quadratic**	r_s	0.160	0.080	0.070	0.120
	p_{fdr}	0.113	0.535	0.576	0.274
**rs2_Right_Y quadratic**	r_s	-0.060	0.000	0.080	-0.020
	p_{fdr}	0.621	0.978	0.502	0.913
**rs2_Right_Z quadratic**	r_s	-0.070	-0.030	-0.120	-0.080
	p_{fdr}	0.571	0.913	0.264	0.540
**rs2_Right_X cubic**	r_s	0.010	-0.030	0.080	0.000

	p_{fdr}	0.967	0.913	0.520	0.978
**rs2_Right_Y cubic**	r_s	0.040	-0.010	-0.020	-0.030
	p_{fdr}	0.849	0.972	0.913	0.913
**rs2_Right_Z cubic**	r_s	-0.120	-0.090	-0.090	-0.100
	p_{fdr}	0.261	0.443	0.436	0.367
N = 147					
**rs3_Right_X Linear**	r_s	0.030	-0.080	-0.110	-0.030
	p_{fdr}	0.771	0.524	0.348	0.771
**rs3_Right_Y Linear**	r_s	0.070	-0.040	-0.080	0.010
	p_{fdr}	0.535	0.721	0.486	0.933
**rs3_Right_Z Linear**	r_s	0.040	0.040	0.120	0.000
	p_{fdr}	0.722	0.722	0.284	0.982
**rs3_Right_X quadratic**	r_s	0.120	0.050	0.080	0.080
	p_{fdr}	0.269	0.686	0.486	0.486
**rs3_Right_Y quadratic**	r_s	-0.070	0.050	0.040	-0.050
	p_{fdr}	0.535	0.686	0.721	0.686
**rs3_Right_Z quadratic**	r_s	-0.080	-0.020	-0.060	-0.050
	p_{fdr}	0.534	0.820	0.648	0.709
**rs3_Right_X cubic**	r_s	0.020	0.070	0.100	0.030
	p_{fdr}	0.832	0.597	0.415	0.798
**rs3_Right_Y cubic**	r_s	-0.150	0.010	0.040	-0.100
	p_{fdr}	0.153	0.912	0.722	0.399
**rs3_Right_Z cubic**	r_s	-0.050	-0.060	-0.080	0.000
	p_{fdr}	0.686	0.648	0.486	0.982
N = 144					
**ΔRight_X Linear**	r_s	0.030	-0.060	-0.060	-0.020
	p_{fdr}	0.849	0.649	0.648	0.898
**ΔRight_Y Linear**	r_s	0.120	-0.090	0.080	0.010
	p_{fdr}	0.319	0.506	0.561	0.964
**ΔRight_Z Linear**	r_s	0.000	0.030	-0.080	-0.060
	p_{fdr}	0.984	0.849	0.561	0.648
**ΔRight_X quadratic**	r_s	-0.060	-0.030	0.000	-0.080
	p_{fdr}	0.660	0.858	0.976	0.561
**ΔRight_Y quadratic**	r_s	-0.060	0.050	-0.120	-0.080
	p_{fdr}	0.648	0.771	0.283	0.561
**ΔRight_Z quadratic**	r_s	0.030	-0.010	0.160	0.100
	p_{fdr}	0.862	0.964	0.119	0.455
**ΔRight_X cubic**	r_s	0.040	0.070	0.030	0.040
	p_{fdr}	0.791	0.589	0.849	0.812
**ΔRight_Y cubic**	r_s	-0.230*	0.050	-0.020	-0.110

	p_{fdr}	0.014	0.702	0.898	0.351
**ΔRight_Z cubic**	r_s	0.010	-0.040	-0.010	0.040
	p_{fdr}	0.964	0.791	0.964	0.791
N = 131					

rs2: pre-stress rs; rs3: post-induction rs; Δ: the difference between pre-stress and post-induction rs;
* p<0.05.

Table S3. Spearman correlations between CA and the first-order gradient parameters after controlling age, gender and medication

		**Emotional Neglect**	**Psychological Abuse**	**Physical Abuse**	**Childhood Trauma**
**rs2_Left_X Linear**	r_s	0.090	0.010	0.150	0.090
	p_{fdr}	0.440	0.899	0.154	0.440
**rs2_Left_Y Linear**	r_s	-0.050	-0.010	-0.070	-0.060
	p_{fdr}	0.662	0.898	0.575	0.650
**rs2_Left_Z Linear**	r_s	0.060	-0.050	-0.050	0.030
	p_{fdr}	0.650	0.670	0.653	0.744
**rs2_Left_X quadratic**	r_s	0.100	0.070	-0.030	0.120
	p_{fdr}	0.403	0.584	0.773	0.297
**rs2_Left_Y quadratic**	r_s	0.080	0.040	0.040	0.080
	p_{fdr}	0.495	0.693	0.709	0.541
**rs2_Left_Z quadratic**	r_s	-0.080	-0.060	-0.050	-0.110
	p_{fdr}	0.495	0.605	0.653	0.352
**rs2_Left_X cubic**	r_s	-0.100	-0.010	-0.120	-0.100
	p_{fdr}	0.393	0.898	0.297	0.393
**rs2_Left_Y cubic**	r_s	0.090	-0.050	0.060	0.040
	p_{fdr}	0.469	0.653	0.641	0.709
**rs2_Left_Z cubic**	r_s	-0.010	-0.050	0.050	-0.070
	p_{fdr}	0.898	0.653	0.663	0.575
N = 148					
**rs3_Left_X Linear**	r_s	0.030	0.060	0.040	0.080
	p_{fdr}	0.794	0.619	0.734	0.429
**rs3_Left_Y Linear**	r_s	0.100	-0.080	-0.090	-0.030
	p_{fdr}	0.361	0.429	0.384	0.747
**rs3_Left_Z Linear**	r_s	0.060	0.050	0.140	0.160
	p_{fdr}	0.569	0.666	0.162	0.095
**rs3_Left_X quadratic**	r_s	0.050	0.040	0.160	0.130
	p_{fdr}	0.666	0.709	0.089	0.197
**rs3_Left_Y quadratic**	r_s	-0.080	0.040	0.030	-0.010
	p_{fdr}	0.429	0.709	0.743	0.908
**rs3_Left_Z quadratic**	r_s	-0.020	-0.040	-0.130	-0.100
	p_{fdr}	0.806	0.709	0.197	0.350

**rs3_Left_X cubic**	r_s	-0.020	-0.110	-0.070	-0.130
	p_{fdr}	0.821	0.331	0.544	0.197
**rs3_Left_Y cubic**	r_s	-0.170	-0.050	0.010	-0.090
	p_{fdr}	0.088	0.666	0.908	0.384
**rs3_Left_Z cubic**	r_s	0.040	-0.090	-0.110	-0.090
	p_{fdr}	0.725	0.384	0.319	0.385
N = 152					
**ΔLeft_X Linear**	r_s	-0.040	0.000	-0.020	0.010
	p_{fdr}	0.800	0.990	0.986	0.990
**ΔLeft_Y Linear**	r_s	0.130	0.000	-0.010	0.040
	p_{fdr}	0.245	0.990	0.986	0.800
**ΔLeft_Z Linear2**	r_s	0.060	0.110	0.080	0.140
	p_{fdr}	0.715	0.327	0.602	0.204
**ΔLeft_X quadratic**	r_s	0.010	-0.040	0.030	0.010
	p_{fdr}	0.990	0.840	0.898	0.990
**ΔLeft_Y quadratic**	r_s	-0.160	-0.070	0.000	-0.110
	p_{fdr}	0.136	0.620	0.990	0.359
**ΔLeft_Z quadratic**	r_s	0.030	0.090	-0.060	0.010
	p_{fdr}	0.898	0.510	0.673	0.986
**ΔLeft_X cubic**	r_s	0.040	-0.010	0.030	-0.010
	p_{fdr}	0.803	0.986	0.898	0.986
**ΔLeft_Y cubic**	r_s	-0.220*	-0.060	-0.060	-0.150
	p_{fdr}	0.021	0.673	0.707	0.155
**ΔLeft_Z cubic**	r_s	-0.020	-0.040	-0.050	-0.050
	p_{fdr}	0.933	0.800	0.760	0.800
N = 136					
**rs2_Right_X Linear**	r_s	-0.040	0.000	-0.020	0.010
	p_{fdr}	0.923	0.895	0.879	0.963
**rs2_Right_Y Linear**	r_s	0.130	0.000	-0.010	0.040
	p_{fdr}	0.361	0.963	0.776	0.765
**rs2_Right_Z Linear**	r_s	0.060	0.110	0.080	0.140
	p_{fdr}	0.627	0.963	0.114	0.726
**rs2_Right_X quadratic**	r_s	0.010	-0.040	0.030	0.010
	p_{fdr}	0.097	0.404	0.355	0.182
**rs2_Right_Y quadratic**	r_s	-0.160	-0.070	0.000	-0.110
	p_{fdr}	0.895	0.972	0.355	0.895
**rs2_Right_Z quadratic**	r_s	0.030	0.090	-0.060	0.010
	p_{fdr}	0.353	0.787	0.072	0.223
**rs2_Right_X cubic**	r_s	0.040	-0.010	0.030	-0.010
	p_{fdr}	0.923	0.879	0.545	0.963
**rs2_Right_Y cubic**	r_s	-0.220	-0.060	-0.060	-0.150
	p_{fdr}	0.396	1.000	0.963	0.895

**rs2_Right_Z cubic**	r_s	-0.020	-0.040	-0.050	-0.050
	p_{fdr}	0.282	0.563	0.412	0.355
N = 147					
**rs3_Right_X Linear**	r_s	0.010	-0.060	-0.080	-0.040
	p_{fdr}	0.949	0.638	0.519	0.730
**rs3_Right_Y Linear**	r_s	0.020	-0.010	-0.070	-0.020
	p_{fdr}	0.865	0.907	0.575	0.865
**rs3_Right_Z Linear**	r_s	0.080	0.030	0.120	0.030
	p_{fdr}	0.489	0.790	0.291	0.804
**rs3_Right_X quadratic**	r_s	0.120	0.060	0.130	0.090
	p_{fdr}	0.291	0.638	0.232	0.459
**rs3_Right_Y quadratic**	r_s	-0.030	0.040	0.020	-0.020
	p_{fdr}	0.790	0.742	0.839	0.843
**rs3_Right_Z quadratic**	r_s	-0.050	-0.050	-0.110	-0.050
	p_{fdr}	0.675	0.681	0.350	0.703
**rs3_Right_X cubic**	r_s	0.060	0.060	0.080	0.060
	p_{fdr}	0.646	0.646	0.519	0.648
**rs3_Right_Y cubic**	r_s	-0.100	-0.030	0.010	-0.090
	p_{fdr}	0.408	0.800	0.906	0.484
**rs3_Right_Z cubic**	r_s	-0.100	-0.050	-0.090	-0.040
	p_{fdr}	0.368	0.689	0.480	0.742
N = 144					
**ΔRight_X Linear**	r_s	-0.020	-0.070	-0.050	-0.060
	p_{fdr}	0.842	0.632	0.740	0.688
**ΔRight_Y Linear**	r_s	0.070	-0.080	0.060	-0.010
	p_{fdr}	0.617	0.616	0.650	0.893
**ΔRight_Z Linear**	r_s	0.030	0.020	-0.060	-0.040
	p_{fdr}	0.784	0.842	0.650	0.775
**ΔRight_X quadratic**	r_s	-0.030	-0.040	-0.020	-0.070
	p_{fdr}	0.812	0.784	0.842	0.632
**ΔRight_Y quadratic**	r_s	-0.050	0.040	-0.160	-0.100
	p_{fdr}	0.697	0.756	0.115	0.446
**ΔRight_Z quadratic**	r_s	0.040	0.010	0.200*	0.130
	p_{fdr}	0.756	0.948	0.046	0.242
**ΔRight_X cubic**	r_s	0.090	0.070	0.020	0.060
	p_{fdr}	0.543	0.632	0.842	0.657
**ΔRight_Y cubic**	r_s	-0.200*	0.050	-0.050	-0.110
	p_{fdr}	0.038	0.733	0.737	0.364
**ΔRight_Z cubic**	r_s	-0.070	-0.020	0.040	0.010
	p_{fdr}	0.632	0.886	0.784	0.893

N = 131

rs2: pre-stress rs; rs3: post-induction rs; Δ: the difference between pre-stress and post-induction rs; * $p<0.05$.

Table S4. Comparing the correlations of emotional neglect with other CA types

		rs3_Left_Y cubic	ΔLeft_Y cubic	ΔRight_Y cubic
**Psychological Abuse**	*z*	-1.575	-1.507	-2.923
	p	0.058	0.066	0.002 **
**Physical Abuse**	*z*	-1.910	-1.438	-1.925
	p	0.028*	0.075	0.027*

rs3: post-induction rs; * $p<0.05$, ** $p<0.01$.

Table S5. Summary of the linear mixed models

	Dependent variables	Fixed factors	Random factors
**Model 1**	Left_Y cubic	Resting-state (repeated measure);<br>Emotional Neglect;<br>Depressive Severity.	Subjects
**Model 2**	Left_Y cubic	Resting-state (repeated measure);<br>Emotional Neglect;<br>Comorbidity	Subjects
**Model 3**	ΔY cubic	Sides of the striatum (repeated measure);<br>Emotional Neglect;<br>Depressive Severity.	Subjects
**Model 4**	ΔY cubic	Sides of the striatum (repeated measure);<br>Emotional Neglect;<br>Comorbidity.	Subjects

Left_Y cubic: Y cubic TSM parameter at the left striatum; ΔY cubic: the changes of Y cubic parameter values between the pre-stress rs and post-stress induction rs.

Table S6. Linear mixed models with emotional neglect, the depressive severity and comorbidity

**Type III Analysis of Variance Table with Satterthwaite's method**						
The model for Y cubic parameter at the left striatum with resting states(rs), emotional neglect and depressive severity						
	Sum Sq	Mean Sq	NumDF	DenDF	F value	Pr(>F)
**rs**	0.000	0.000	1	129.530	0.461	0.499
**Emotional Neglect**	0.001	0.001	1	133.450	0.595	0.442
**Depressive Severity**	0.000	0.000	1	136.820	0.012	0.915
**rs:Emotional Neglect**	0.000	0.000	1	121.980	0.364	0.547

**rs:Depressive Severity**	0.000	0.000	1	125.640	0.410	0.523
**Emotional Neglect: Depressive Severity**	0.001	0.001	1	132.160	0.820	0.367
**rs:Emotional Neglect: Depressive Severity**	0.000	0.000	1	120.500	0.022	0.883
The model for Y cubic parameter at the left striatum with rs, emotional neglect and comorbidity						
	Sum Sq	Mean Sq	NumDF	DenDF	F value	Pr(>F)
**rs**	0.002	0.002	1	140.890	1.856	0.175
**Emotional Neglect**	0.001	0.001	1	145.570	0.845	0.360
**Comorbidity**	0.001	0.001	1	147.850	0.703	0.403
**rs:Emotional Neglect**	0.002	0.002	1	133.160	1.782	0.184
**rs: Comorbidity**	0.002	0.002	1	135.010	2.481	0.118
**Emotional Neglect: Comorbidity**	0.002	0.002	1	142.910	1.626	0.204
**rs:Emotional Neglect: Comorbidity**	0.005	0.005	1	129.940	5.172	0.025 *
The model for the difference of Y cubic parameter between the two resting states with sides of the striatum (left/right), emotional neglect and depressive severity						
	Sum Sq	Mean Sq	NumDF	DenDF	F value	Pr(>F)
**Side**	0.000	0.000	1	110.900	0.269	0.605
**Emotional Neglect**	0.000	0.000	1	111.940	0.082	0.775
**Depressive Severity**	0.000	0.000	1	117.120	0.174	0.678
**Side: Emotional Neglect**	0.005	0.005	1	106.410	2.765	0.099
**Side: Depressive Severity**	0.000	0.000	1	111.750	0.108	0.743
**Emotional Neglect: Depressive Severity**	0.001	0.001	1	113.420	0.732	0.394
**Side: Emotional Neglect: Depressive Severity**	0.005	0.005	1	108.000	2.712	0.102
The model for the difference of Y cubic parameter between the two resting states with sides of the striatum (left/right), emotional neglect and comorbidity						
	Sum Sq	Mean Sq	NumDF	DenDF	F value	Pr(>F)
**Side**	0.001	0.001	1	121.220	0.613	0.435
**Emotional Neglect**	0.001	0.001	1	123.090	0.386	0.535
**Comorbidity**	0.005	0.005	1	129.920	2.806	0.096
**Side: Emotional Neglect**	0.000	0.000	1	118.020	0.283	0.596
**Side: Comorbidity**	0.000	0.000	1	125.260	0.095	0.758
**Emotional Neglect: Comorbidity**	0.007	0.007	1	127.670	3.985	0.048 *
**Side: Emotional**	0.000	0.000	1	122.580	0.083	0.773

Neglect: Comorbidity

* p<0.05

Table S7. Moderating models with emotional neglect and comorbidity

Model: the interaction between emotional neglect and comorbidity for Y cubic parameter at the left striatum of pre-stress rs

**Model Summary**	**R**	**R-sq**	**MSE**	**F**	**df1**	**df2**	**p**
	0.062	0.004	0.002	0.186	3	143	0.906
	**coeff**	**se**	**t**	**p**	**LLCI**	**ULCI**	
**constant**	-0.031	0.010	-3.047	0.003**	-0.051	-0.011	
**Emotional Neglect**	-0.004	0.008	-0.582	0.562	-0.019	0.011	
**Comorbidity**	-0.002	0.005	-0.318	0.751	-0.012	0.009	
**Interaction**	0.002	0.003	0.664	0.508	-0.005	0.009	

Test(s) of highest order unconditional interaction(s):

	**R^2-chng**	**F**	**df1**	**df2**	**p**
**Emotional Neglect * comorbidity**	0.003	0.440	1	143	0.508

Model: the interaction between emotional neglect and comorbidity for Y cubic parameter at the left striatum of post-induction rs

**Model Summary**	**R**	**R-sq**	**MSE**	**F**	**df1**	**df2**	**p**
	0.204	0.042	0.002	2.122	3	147	0.100
	**coeff**	**se**	**t**	**p**	**LLCI**	**ULCI**	
**constant**	-0.047	0.011	-4.356	0.000**	-0.069	-0.026	
**Emotional Neglect**	0.011	0.008	1.362	0.175	-0.005	0.026	
**Comorbidity**	0.008	0.005	1.403	0.163	-0.003	0.018	
**Interaction**	-0.008	0.004	-2.198	0.030*	-0.015	-0.001	

Test(s) of highest order unconditional interaction(s):

	**R^2-chng**	**F**	**df1**	**df2**	**p**
**Emotional Neglect * comorbidity**	0.032	4.831	1	147	0.030*

Conditional effects of emotional neglect at values of the moderator (comorbidity):

**Comorbidity**	**Effect**	**se**	**t**	**p**	**LLCI**	**ULCI**
1	0.003	0.005	0.558	0.577	-0.007	0.013
2	-0.005	0.004	-1.175	0.242	-0.013	0.003
3	-0.013	0.006	-2.212	0.029*	-0.024	-0.001

Model: the interaction between emotional neglect and comorbidity for the difference of Y cubic

parameter between the two resting states.

Model Summary	R	R-sq	MSE	F	df1	df2	p
	0.196	0.038	0.002	3.457	3	261	0.017*
	**coeff**	**se**	**t**	**p**	**LLCI**	**ULCI**	
**constant**	-0.008	0.008	-0.997	0.320	-0.024	0.008	
**Emotional Neglect**	0.003	0.006	0.553	0.581	-0.008	0.015	
**Comorbidity**	0.007	0.004	1.794	0.074	-0.001	0.015	
**Interaction**	-0.006	0.003	-2.132	0.034*	-0.011	0.000	

Test(s) of highest order unconditional interaction(s):

	R^2-chng	F	df1	df2	p	
**Emotional Neglect * comorbidity**	0.017	4.546	1	261	0.034*	

Conditional effects of emotional neglect at values of the moderator (comorbidity):

Comorbidity	Effect	se	t	p	LLCI	ULCI
1	-0.003	0.004	-0.649	0.517	-0.010	0.005
2	-0.008	0.003	-2.637	0.009**	-0.015	-0.002
3	-0.014	0.004	-3.191	0.002**	-0.023	-0.005

* *p*<0.05, ***p*<0.01.

Table S8. Linear mixed models with emotional neglect, the depressive severity and comorbidity (controlled by age, gender and medication)

Type III Analysis of Variance Table with Satterthwaite's method

The model for Y cubic parameter at the left striatum with resting states(rs), emotional neglect and depressive severity

	Sum Sq	Mean Sq	NumDF	DenDF	F value	Pr(>F)
**rs**	0.000	0.000	1	128.260	0.385	0.536
**Emotional Neglect**	0.001	0.001	1	129.860	1.085	0.300
**Depressive Severity**	0.000	0.000	1	133.600	0.003	0.954
**rs:Emotional Neglect**	0.000	0.000	1	120.000	0.415	0.521
**rs:Depressive Severity**	0.000	0.000	1	124.010	0.336	0.564
**Emotional Neglect: Depressive Severity**	0.001	0.001	1	128.430	1.037	0.311
**rs:Emotional Neglect: Depressive Severity**	0.000	0.000	1	118.390	0.040	0.841

The model for Y cubic parameter at the left striatum with rs, emotional neglect and comorbidity

	Sum Sq	Mean Sq	NumDF	DenDF	F value	Pr(>F)
**rs**	0.002	0.002	1	140.120	1.598	0.208
**Emotional Neglect**	0.001	0.001	1	142.730	1.266	0.262
**Comorbidity**	0.000	0.000	1	145.160	0.003	0.959

**rs:Emotional Neglect**	0.002	0.002	1	131.820	1.717	0.192
**rs: Comorbidity**	0.002	0.002	1	133.850	2.220	0.139
**Emotional Neglect: Comorbidity**	0.001	0.001	1	139.840	1.223	0.271
**rs:Emotional Neglect: Comorbidity**	0.005	0.005	1	128.400	5.063	0.026*
The model for the difference of Y cubic parameter between the two resting states with sides of the striatum (left/right), emotional neglect and depressive severity						
	Sum Sq	Mean Sq	NumDF	DenDF	F value	Pr(>F)
**Side**	0.001	0.001	1	110.410	0.301	0.585
**Emotional Neglect**	0.000	0.000	1	110.920	0.012	0.914
**Depressive Severity**	0.000	0.000	1	116.150	0.207	0.650
**Side: Emotional Neglect**	0.004	0.004	1	105.880	2.611	0.109
**Side: Depressive Severity**	0.000	0.000	1	111.250	0.121	0.729
**Emotional Neglect: Depressive Severity**	0.001	0.001	1	112.420	0.478	0.491
**Side: Emotional Neglect: Depressive Severity**	0.004	0.004	1	107.470	2.578	0.111
The model for the difference of Y cubic parameter between the two resting states with sides of the striatum (left/right), emotional neglect and comorbidity						
	Sum Sq	Mean Sq	NumDF	DenDF	F value	Pr(>F)
**Side**	0.001	0.001	1	120.590	0.642	0.425
**Emotional Neglect**	0.001	0.001	1	121.740	0.494	0.484
**Comorbidity**	0.007	0.007	1	128.650	3.948	0.049*
**Side: Emotional Neglect**	0.001	0.001	1	117.370	0.317	0.575
**Side: Comorbidity**	0.000	0.000	1	124.640	0.090	0.764
**Emotional Neglect: Comorbidity**	0.008	0.008	1	126.360	4.732	0.031*
**Side: Emotional Neglect: Comorbidity**	0.000	0.000	1	121.960	0.092	0.763

* $p<0.05$

Table S9. Moderating models with emotional neglect and comorbidity (controlled by age, gender and medication use)

Model: the interaction between emotional neglect and comorbidity for Y cubic parameter at the left striatum of pre-stress rs							
**Model Summary**	**R**	**R-sq**	**MSE**	**F**	**df1**	**df2**	**p**
	0.097	0.009	0.002	0.448	3	143	0.719
	**coeff**	**se**	**t**	**p**	**LLCI**	**ULCI**	
**constant**	0.009	0.010	0.892	0.374	-0.011	0.028	

**Emotional Neglect**	-0.003	0.007	-0.366	0.715	-0.017	0.012	
**Comorbidity**	-0.005	0.005	-1.060	0.291	-0.015	0.005	
**Interaction**	0.003	0.003	0.830	0.408	-0.004	0.009	

Test(s) of highest order unconditional interaction(s):

	**R^2-chng**	**F**	**df1**	**df2**	**p**
**Emotional Neglect * comorbidity**	0.005	0.689	1	143	0.408

Model: the interaction between emotional neglect and comorbidity for Y cubic parameter at the left striatum of post-induction rs

**Model Summary**	**R**	**R-sq**	**MSE**	**F**	**df1**	**df2**	**p**
	0.221	0.049	0.002	2.518	3	147	0.060
	**coeff**	**se**	**t**	**p**	**LLCI**	**ULCI**	
**constant**	-0.006	0.011	-0.543	0.588	-0.026	0.015	
**Emotional Neglect**	0.011	0.008	1.502	0.135	-0.004	0.026	
**Comorbidity**	0.003	0.005	0.603	0.548	-0.007	0.013	
**Interaction**	-0.007	0.003	-2.031	0.044*	-0.014	0.000	

Test(s) of highest order unconditional interaction(s):

	**R^2-chng**	**F**	**df1**	**df2**	**p**
**Emotional Neglect * comorbidity**	0.027	4.125	1	147	0.044*

Conditional effects of emotional neglect at values of the moderator (comorbidity):

**Comorbidity**	**Effect**	**se**	**t**	**p**	**LLCI**	**ULCI**
1	0.004	0.005	0.886	0.377	-0.006	0.014
2	-0.003	0.004	-0.626	0.532	-0.011	0.005
3	-0.010	0.006	-1.709	0.090	-0.020	0.002

Model: the interaction between emotional neglect and comorbidity for the difference of Y cubic parameter between the two resting states.

**Model Summary**	**R**	**R-sq**	**MSE**	**F**	**df1**	**df2**	**p**
	0.205	0.042	0.002	3.827	3	261	0.010*
	**coeff**	**se**	**t**	**p**	**LLCI**	**ULCI**	
**constant**	-0.005	0.008	-0.568	0.571	-0.020	0.011	
**Emotional Neglect**	0.004	0.006	0.639	0.524	-0.008	0.015	
**Comorbidity**	0.009	0.004	2.099	0.037	0.001	0.017	
**Interaction**	-0.006	0.003	-2.299	0.022*	-0.012	-0.001	

**Test(s) of highest order unconditional interaction(s):**							
	**R^2-chng**	**F**	**df1**	**df2**	**p**		
**Emotional Neglect * comorbidity**	0.019	5.284	1	261	0.022*		
**Conditional effects of emotional neglect at values of the moderator (comorbidity):**							
**Comorbidity**	**Effect**	**se**	**t**	**p**	**LLCI**	**ULCI**	
1	-0.002	0.004	-0.634	0.527	-0.010	0.005	-0.002
2	-0.009	0.003	-2.763	0.006**	-0.015	-0.003	-0.009
3	-0.015	0.004	-3.384	0.001**	-0.024	-0.006	-0.015

* *p*<0.05, ***p*<0.01.
